# In Vitro Evidence for the Dual Antioxidant and Anti-Inflammatory Roles of *Hypericum triquetrifolium* in Cancer Therapy: Selective Cytotoxicity Against Pancreatic Cancer Cells

**DOI:** 10.3390/molecules31101628

**Published:** 2026-05-12

**Authors:** Ece Sabuncu, Yağmur Özhan, Dilara Güreşçi, Gizem Inetas Yengin, Merve Güdül Bacanlı, Etil Güzelmeriç, Dilek Telci, Hande Sipahi

**Affiliations:** 1 Department of Toxicology, Faculty of Pharmacy, Yeditepe University, 34755 İstanbul, Türkiye; ece.sabuncu@gmail.com (E.S.); yagmur.ozhan@sbu.edu.tr (Y.Ö.); dilaraguresci@gmail.com (D.G.); 2 Department of Toxicology, Hamidiye Faculty of Pharmacy, University of Health Sciences Turkey, 34668 İstanbul, Türkiye; 3 Department of Genetics and Bioengineering, Faculty of Engineering, Yeditepe University, 34755 İstanbul, Türkiye; gizeminetas@gmail.com (G.I.Y.); dilek.telci@yeditepe.edu.tr (D.T.); 4 Department of Pharmaceutical Toxicology, Gülhane Faculty of Pharmacy, University of Health Sciences Turkey, 06018 Ankara, Türkiye; merve.gudulbacanli@sbu.edu.tr; 5 Health Institutes of Türkiye (TÜSEB), Turkish Vaccine Institute, 06270 Ankara, Türkiye; 6 Department of Pharmacognosy, Faculty of Pharmacy, Yeditepe University, 34755 İstanbul, Türkiye; etil.ariburnu@yeditepe.edu.tr

**Keywords:** *Hypericum triquetrifolium*, anticancer, anti-inflammation, oxidative stress, apoptosis, genotoxicity

## Abstract

*Hypericum* species are known for their phytochemical richness and diverse pharmacological activities; however, the biological potential and underlying mechanisms of *Hypericum triquetrifolium* remain unclear. This study aimed to investigate the phytochemical profile and bioactivities of *Hypericum triquetrifolium* hydroalcoholic extract (HTE). Plant samples collected from Gelibolu (Çanakkale, Türkiye) were analyzed by HPTLC, revealing the presence of rutin, hyperoside, chlorogenic acid, neochlorogenic acid, and hypericin. Antioxidant activity was evaluated using DPPH, CUPRAC, and FRAP assays, demonstrating strong activity across all tests. Anti-inflammatory effects were assessed in LPS-stimulated RAW264.7 macrophages. HTE significantly reduced nitrite, PGE_2_, IL-6, ROS, and MDA levels, with the most pronounced effect observed at 0.5 mg/mL (*p* < 0.05). Cytotoxic and anticancer activities were evaluated in MIA PaCa-2 pancreatic cancer cells and healthy human dermal fibroblasts. The extract exhibited selective cytotoxicity toward cancer cells (IC_50_ = 0.16 mg/mL), with approximately fivefold lower toxicity in healthy cells. Cell cycle analysis demonstrated G0/G1 arrest, while the Annexin V assay revealed dose-dependent apoptosis (*p* < 0.0001). DNA damage analysis further supported its anticancer potential. Overall, HTE exhibits potent antioxidant, anti-inflammatory, and selective anticancer activities by reducing oxidative stress, inducing cell cycle arrest, and promoting apoptosis, highlighting its potential as a therapeutic agent.

## 1. Introduction

*Hypericum* species have been widely used to treat wounds, burns, and inflammatory conditions. Although ethnobotanical data on *Hypericum triquetrifolium* are limited, its reported wound healing and anti-inflammatory activities support its traditional medicinal relevance. *Hypericum triquetrifolium* demonstrated significant anti-inflammatory activity in a carrageenan-induced inflammation model in rats [1]. Intraperitoneal administration of the extract (25, 50, and 60 mg/kg) significantly reduced paw edema in a dose-dependent manner. In addition, *H. triquetrifolium* ointment (5%) showed remarkable wound healing properties [2].

*Hypericum triquetrifolium*, a medicinal plant native to the Mediterranean region, has garnered considerable interest owing to its rich phytochemical profile and diverse pharmacological properties. Notably, it contains high levels of bioactive compounds such as hypericin, hyperforin, and pseudohypericin, which exhibit potent antioxidant and anti-inflammatory activities [3,4]. Compared to the widely studied *Hypericum perforatum* of the same genus, *H. triquetrifolium* reportedly harbors higher concentrations of these constituents, potentially enhancing its therapeutic efficacy [3]. Previous studies have demonstrated that methanolic extracts of *H. triquetrifolium* inhibit human leukocyte myeloperoxidase (MPO), a key enzyme involved in inflammatory processes, thereby preventing the formation of reactive oxidants such as hypochlorous acid and reducing oxidative tissue damage [5].

Chronic inflammation and oxidative stress play pivotal roles in cancer initiation and progression. Inflammatory processes drive the production of reactive oxygen species (ROS), which damage cellular components such as DNA, proteins, and lipids, thereby promoting tumorigenesis [6]. Although endogenous antioxidant defense systems, such as superoxide dismutase (SOD), glutathione (GSH), and catalase (CAT), function to neutralize ROS, excessive oxidative stress can overwhelm these protective mechanisms and lead to chronic pathological conditions, including cancer [7]. Given its reported ability to modulate inflammatory mediators, including tumor necrosis factor-alpha (TNF-α) and nitric oxide (NO), *H. triquetrifolium* may represent a potential therapeutic candidate for mitigating the oxidative and inflammatory microenvironment associated with cancer development [8].

Although several *Hypericum* species, including *H. perforatum*, *H. brasiliense*, and *H. triquetrifolium*, have demonstrated antiproliferative activity against various cancer cell lines including MDA-MB-231, HepG2, A549, and PC3 cells [9], the anticancer potential of *H. triquetrifolium* remains insufficiently characterized. Variations in phytochemical composition among *Hypericum* species may lead to differences in pharmacological effects [10,11,12]. Furthermore, its effects on pancreatic cancer models and advanced three-dimensional (3D) tumor systems have not yet been investigated.

Therefore, this study systematically investigated the antioxidant, anti-inflammatory, and anticancer properties of *H. triquetrifolium* extract (HTE), with a particular focus on pancreatic cancer models using advanced 3D tumor spheroid systems that better recapitulate the tumor microenvironment. The study further elucidates the underlying mechanisms by examining oxidative stress modulation, inflammatory mediator regulation, cell cycle dynamics, apoptosis induction, and selective cytotoxic effects between cancerous and non-cancerous cells. By integrating conventional 2D cultures with physiologically relevant 3D models, this work addresses key gaps in understanding the plant’s biological and therapeutic potential. Ultimately, these findings provide mechanistic insight into the pharmacological activities of *H. triquetrifolium* and contribute to understanding its effects on oxidative stress, inflammation, and cancer-related cellular processes.

## 2. Results

### 2.1. Phytochemical Analysis by HPTLC and TPC of H. triquetrifolium

**Figure 1:**
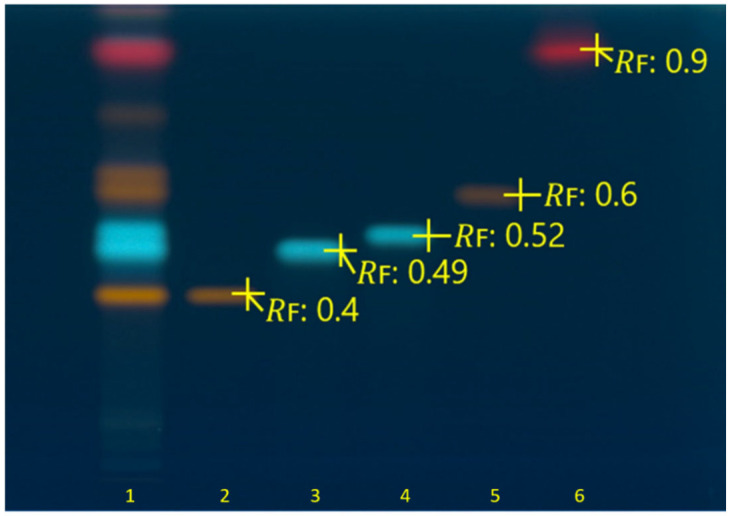
HPTLC chromatogram of *H. triquetrifolium* hydroalcoholic extract and standards on silica gel F_254_ plate captured at 366 nm after derivatization with NP and PEG reagents. 1: *H. triquetrifolium* extract (20 µg/band), 2: rutin (0.3 µg/band), 3: chlorogenic acid (0.2 µg/band), 4: neochlorogenic acid (0.2 µg/band), 5: hyperoside (0.3 µg/band), and 6: hypericin (0.01 µg/band). NP: natural product. PEG: polyethylene glycol.

The chemical fingerprint of *H. triquetrifolium* was comparatively analyzed with known standard solutions and their retardation factors (*R*_F_), as demonstrated in Figure 1. Accordingly, rutin and hyperoside were detected as orange-colored bands at *R*_F_ ≈ 0.4 and *R*_F_ ≈ 0.6, respectively. Chlorogenic acid with an *R*_F_ value of ≈ 0.49 and a neochlorogenic acid with an *R*_F_ ≈ 0.52 were observed in blue-colored bands. A red-colored band corresponding to hypericin was detected at *R*_F_ ≈ 0.9 (Figure 1). Comparison of the sample with the standard bands demonstrated that these standard compounds were observed at the same *R*_F_ values and colors in the sample.

### 2.2. Total Phenolic Content of Extract

The TPC (total phenolic content) of the *H. triquetrifolium* extract was found to be 382.55 ± 15.13 mg GAE/g. This high concentration of phenolic compounds likely contributes to the substantial antioxidant effects observed.

### 2.3. Antioxidant Properties of Extract

Antioxidant activity was determined by using DPPH, CUPRAC and FRAP methods (Table 1). Furthermore, the chemical content was characterized by its phenolic content. This characterization supported further analysis of the extract. Revelation of the bioactive content allowed for continuation of the extraction process.

### 2.4. Anti-Inflammatory Activity

#### 2.4.1. Cytotoxicity and Nitric Oxide Inhibition in LPS-Stimulated RAW264.7 Cells

Prior to evaluating nitrite inhibition as an indicator of anti-inflammatory activity, the cytotoxicity of *H. triquetrifolium* extract in RAW264.7 cells was assessed. This step was performed to distinguish potential anti-inflammatory effects from cytotoxic responses and to ensure that observed changes in nitrite production were not attributable to extract-induced cell death. As shown in Table 2, HTE at a concentration of 1 mg/mL was found to be cytotoxic; therefore, non-cytotoxic concentrations were selected for subsequent analyses.

HTE treatment significantly affected cell viability and nitrite production in LPS-stimulated RAW264.7 cells (Table 2). LPS stimulation markedly increased nitrite levels compared to the control group, confirming the induction of an inflammatory response. Treatment with *H. triquetrifolium* extract resulted in a dose-dependent reduction in nitrite production. Among the tested concentrations, 0.5 mg/mL HTE exhibited the strongest anti-inflammatory activity, reducing nitrite levels to 6.89 ± 0.93 μM and achieving 88% nitrite inhibition. Similarly, 0.25 mg/mL and 0.125 mg/mL concentrations showed substantial inhibition rates of 82% and 76%, respectively.

The inhibitory effect of HTE surpassed that of the positive controls, L-NAME and indomethacin (inhibition rates of 45% and 51%, respectively). However, the highest tested concentration (1 mg/mL) significantly reduced cell viability, indicating cytotoxicity, and was thus excluded from further anti-inflammatory evaluations. Overall, these results demonstrate that *H. triquetrifolium* extract exhibits strong anti-inflammatory activity in LPS-induced RAW264.7 macrophages by significantly suppressing nitrite production at non-cytotoxic concentrations; the highest such concentration (0.5 mg/mL) was used for subsequent PGE_2_ and IL-6 analyses.

#### 2.4.2. Anti-Inflammatory Activity by PGE_2_ Inhibition Assay

**Figure 2:**
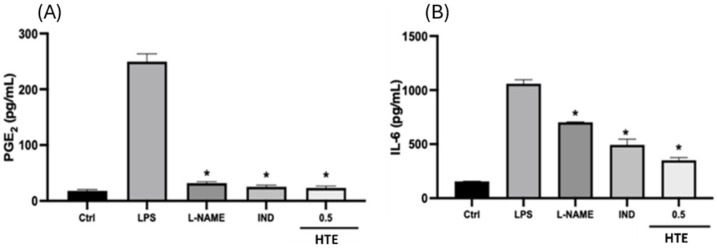
The effects of *H. triquetrifolium* on (**A**) PGE_2_ levels and (**B**) IL-6 levels in LPS-stimulated RAW264.7 cells. The value of 0.5 mg/mL is the highest non-cytotoxic effective concentration. Control: control group with DMEM; LPS: control group stimulated with LPS only; LPS: lipopolysaccharide from *E. coli*; IND: Indomethacin (100 μM); L-NAME: N(gamma)-nitro-L-arginine methyl ester; PGE_2_: prostaglandin E_2_; IL-6: interleukin-6. Statistically significant differences for each compound are indicated against LPS (* *p* < 0.05).

PGE_2_ levels in LPS-stimulated RAW264.7 cells are presented in Figure 2A. LPS stimulation markedly increased PGE_2_ production compared to the control group, confirming the induction of an inflammatory response. Treatment with *H. triquetrifolium* extract (0.5 mg/mL) significantly reduced PGE_2_ levels compared to the LPS group (*p* < 0.05). The reduction observed with HTE was comparable to the effects of the positive controls, L-NAME and indomethacin, both of which also significantly suppressed PGE_2_ production.

#### 2.4.3. Anti-Inflammatory Effect by IL-6 Inhibition Assay

IL-6 levels in LPS-stimulated RAW264.7 cells are shown in Figure 2B. LPS stimulation significantly increased IL-6 production compared to the control group, confirming the induction of an inflammatory response. Treatment with *H. triquetrifolium* extract (0.5 mg/mL) significantly reduced IL-6 levels compared to the LPS group (*p* < 0.05). The extract demonstrated a stronger inhibitory effect than the positive controls, L-NAME and indomethacin, which also significantly decreased IL-6 production. These results indicate that *H. triquetrifolium* extract effectively suppresses LPS-induced inflammatory responses by reducing IL-6 secretion in RAW264.7 macrophages.

#### 2.4.4. Protective Effect of *H. triquetrifolium* Against LPS-Induced Oxidative Stress in RAW264.7 Cells

Extract concentrations (0.0625 and 0.125 mg/mL) showing the highest cell viability were selected for further experiments, as these concentrations also demonstrated strong anti-inflammatory activity in nitrite inhibition assays. Oxidative stress was induced by LPS treatment, and cell lysates were prepared for MDA analysis as a marker of lipid peroxidation. As shown in Table 3, LPS stimulation significantly increased MDA levels compared to the control group, indicating enhanced oxidative stress. Treatment with *H. triquetrifolium* extract markedly reduced MDA levels relative to the LPS group, demonstrating its protective effect against lipid peroxidation and oxidative damage in RAW264.7 cells.

#### 2.4.5. Effect of ROS Activity on LPS-Induced RAW264.7 Cells After HTE Extraction

Intracellular ROS production was analyzed via DCFDA (Dichlorodihydrofluorescein diacetate) staining to evaluate the antioxidant effect of HTE on LPS-induced RAW264.7 cells. To provide a comparative analysis, anti-inflammatory agents such as L-NAME and indomethacin were included as reference controls.

**Figure 3:**
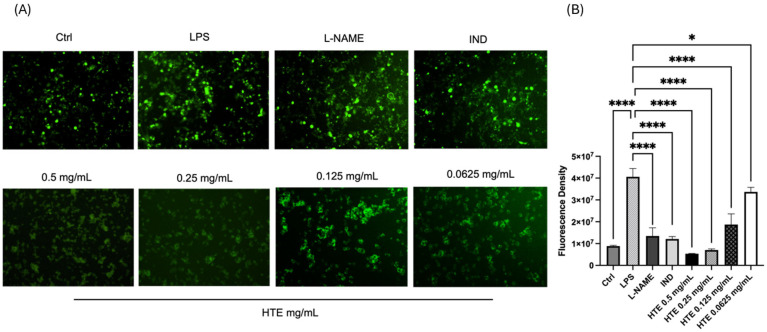
ROS analysis of RAW264.7 cells following *H. triquetrifolium* extract (HTE) treatment. (**A**) Representative immunocytochemistry images for ROS production in LPS-induced cells under indicated experimental conditions (Images captured via Cytell at 10× magnification). (**B**) Statistical analysis of fluorescence intensity. Fluorescence levels were quantified using ImageJ software, and statistical significance was determined via one-way ANOVA followed by Tukey’s post hoc test (GraphPad Prism). Data represent the mean ± SD; * *p* < 0.05 indicates statistical significance compared to the LPS-only group, **** *p* < 0.0001.

In Figure 3A, a concentration-dependent increase in fluorescence intensity is observed as HTE concentrations decreased from 0.5 mg/mL to 0.0625 mg/mL, indicating a reduction in antioxidant efficacy at lower concentrations. Quantitative analysis of the fluorescence intensity was conducted using ImageJ 1.54 software. Statistical significance was determined via one-way ANOVA, followed by Tukey’s post hoc test, which confirmed that HTE treatment significantly reduced ROS production in a dose-dependent manner (*p* < 0.001). In these analyses, all treated groups were compared against the LPS-only induced group to assess relative antioxidant capacity (Figure 3B), with a general significance threshold set at *p* < 0.05.

### 2.5. Potential Anticancer Activity of H. triquetrifolium

#### 2.5.1. Effect on Cell Viability in MIA PaCa-2 Pancreatic Cancer and HDF Cells

The anticancer activity of *H. triquetrifolium* was first assessed on HDF and MIA PaCa-2 cell monolayers prior to the 3D spheroid model. The cytotoxic effects of the extracts differed markedly between healthy human dermal fibroblasts and pancreatic cancer cells. The extracts exhibited selective cytotoxicity against the MIA PaCa-2 cell line, while maintaining at least 70% viability in HDF cells. In 2D cytotoxicity assays, the extracts reduced viability in a dose-dependent manner, with over 70% viability retained in HDF even at the highest concentration. Notably, at 0.125 mg/mL, *H. triquetrifolium* extracts reduced MIA PaCa-2 viability more than in healthy cells, confirming their selectivity for pancreatic cancer cells. The IC_50_ for HDF (Table 4) was 0.68 ± 0.02 mg/mL, vs. 0.16 ± 0.17 mg/mL for MIA PaCa-2—nearly 5-fold higher in HDF—suggesting a selective cytotoxic mechanism with therapeutic potential.

#### 2.5.2. Three-Dimensional (3D) Spheroid Formation/Growth Assay

**Figure 4:**
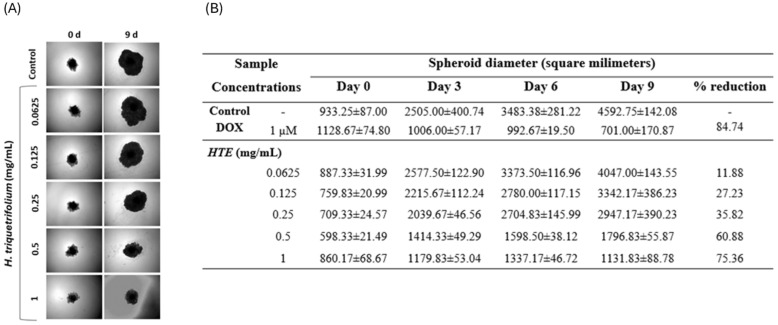
(**A**) Effects of *H. triquetrifolium* extracts on growth of MIA PaCa-2 and HDF cell spheroids. (Magnification 4×) (**B**) Effect of *H. triquetrifolium* extract on spheroid diameters in MIA PaCa-2 cell lines. C: control group with DMEM; DOX: doxorubicin (1 μM). HTE: *H. triquetrifolium* extract.

The findings in 3D models closely resemble in vivo data compared to those in 2D models. Although the anticancer activity of *H. triquetrifolium* has been investigated, its cytotoxic effects on MIA PaCa-2 pancreatic cancer cells and its impact in 3D spheroid models have not been extensively studied (Figure 4). In 2D cytotoxicity assays, 1 mg/mL of *H. triquetrifolium* showed potent anticancer activity compared to other applied concentrations tested concentrations. Spheroids treated with different concentrations of *H. triquetrifolium* showed a significant reduction in size in terms of diameter and area, while control spheroids continued to increase in size for up to 10 days. After 9 days of treatment, spheroids treated with 1 mg/mL *H. triquetrifolium* extract decreased in size by approximately 75% compared to the control (Figure 4). HTE inhibited MIA PaCa-2 and HDF spheroid growth in a dose-dependent manner. Even at the lowest concentration (0.125 mg/mL), HTE reduced the growth rate of spheroids compared to the medium control. At 1 mg/mL, *H. triquetrifolium* extract exhibited a strong inhibitory effect on MIA PaCa-2 and HDF spheroid growth after 9 days. DOX, used as a reference compound, slowed spheroid growth by 84% compared to the control after 9 days of treatment at a concentration of 1 μM. While 0.0625 mg/mL showed no effect, 1 mg/mL exhibited a reduction comparable to that of DOX. Spheroid images were captured every 3 days, as shown in Figure 4.

#### 2.5.3. Cell Cycle Effects of *H. triquetrifolium* in MIA PaCa-2 Cells

**Figure 5:**
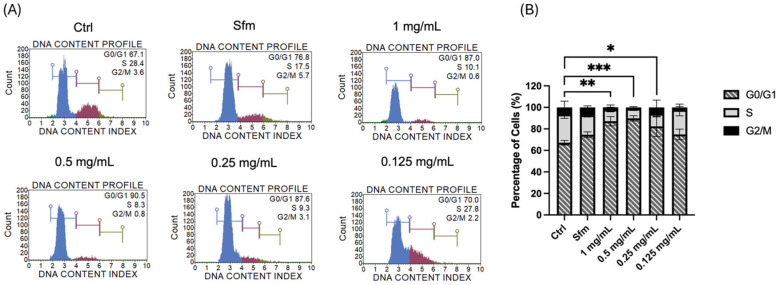
Cell cycle analysis of MIA PaCa-2 cells following *H. triquetrifolium* extract (HTE) treatment. (**A**) Representative histograms illustrating the DNA content profile and cell cycle phase distribution, obtained using the Muse Cell Analyzer. (**B**) Quantitative analysis of the percentage of cells in G0/G1, S, and G2/M phases. Statistical analysis was performed using one-way ANOVA followed by Tukey’s post hoc test (GraphPad Prism 10). Data are presented as the mean ± SD (*n* = 3), and statistically significant differences are indicated by asterisks (* *p* < 0.05, ** *p* < 0.01, and *** *p* < 0).

The cell cycle analysis of MIA PaCa-2 cells treated with HTE revealed a clear, dose-dependent alteration in phase distribution, primarily characterized by arrest in the G0/G1 phase (Figure 5). In untreated control cells, the distribution reflected active proliferation, with representative proportions of 67.1% in G0/G1, 28.4% in S, and 3.6% in G2/M phases (Figure 5A).

Across all independent experiments, HTE treatment induced a consistent and significant accumulation of cells in the G0/G1 phase. Notably, HTE concentrations ranging from 0.25 mg/mL to 1 mg/mL resulted in the most pronounced G0/G1 arrest; specifically, treatment with 0.5 mg/mL HTE resulted in a mean G0/G1 population of 89.81 ± 2.43%, representing a substantial increase compared to the basal levels observed in the control group (Figure 5B). This effect exceeded the levels observed in the serum-free medium (Sfm) group (mean 74.56 ± 2.69%), suggesting that the anti-proliferative impact of the extract is more potent than nutrient deprivation alone. The observed accumulation was accompanied by a marked, dose-dependent decrease in the S and G2/M populations. While the changes in the G0/G1 phase were statistically significant (*p* < 0.05), the reductions in the S and G2/M populations did not reach statistical significance across all concentrations. This further highlights the G0/G1 checkpoint as the primary target of HTE-induced growth inhibition, supporting its potential as an anti-proliferative agent through targeted cell cycle disruption.

#### 2.5.4. Assessment of Apoptosis in MIA PaCa-2 Cells Treated with *H. triquetrifolium*

**Figure 6:**
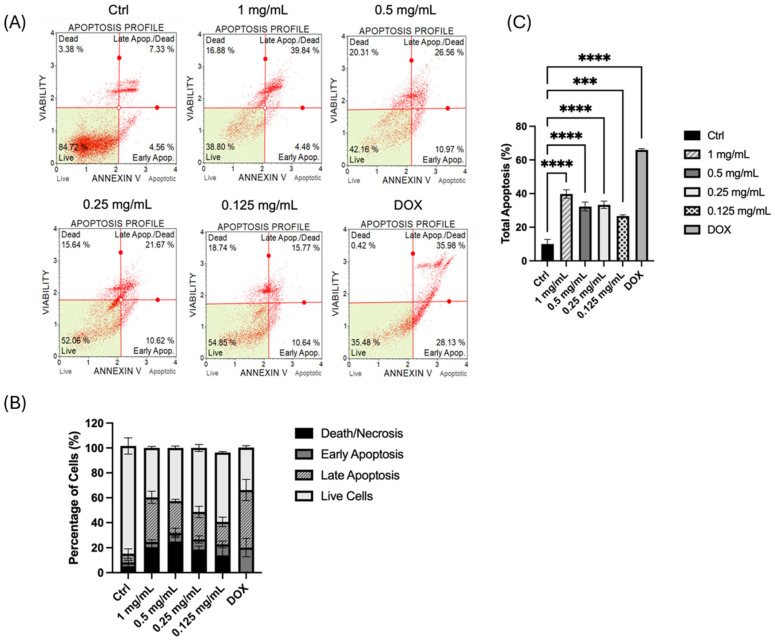
Apoptosis analysis of MIA PaCa-2 cells treated with *H. triquetrifolium*. (**A**) Representative flow cytometry quadrants illustrating the distribution of live (lower left), early apoptotic (lower right), late apoptotic (upper right), and dead (upper left) cell populations, obtained using the Muse Cell Analyzer with Annexin V & Cell Dead staining. (**B**) Quantitative distribution of MIA PaCa-2 cells across different apoptotic stages following 24 h treatment with HTE and doxorubicin (DOX). (**C**) The percentage of total apoptosis (early + late apoptotic cells). Statistical analysis was performed using one-way ANOVA followed by Tukey’s post hoc test (GraphPad Prism 10). Data are presented as the mean ± SD (*n* = 3). Statistically significant differences compared to the untreated control are indicated by asterisks: *** *p* < 0.001 and **** *p* < 0.0001.

The pro-apoptotic effect of HTE on MIA PaCa-2 cells was evaluated via the Muse Annexin V and Cell Dead assay. As shown in the representative apoptosis histograms (Figure 6A), untreated control cells exhibited high viability (~84.7%) with minimal basal apoptotic activity (<12%). In contrast, treatment with HTE for 24 h induced a clear, dose-dependent shift toward programmed cell death. At the highest concentration (1 mg/mL), a sharp increase in the late apoptotic population was observed in the representative plots, with viable cells dropping to approximately 38.8%. Similarly, at 0.5 mg/mL, a substantial proportion of cells transitioned into late apoptosis. Moderate apoptotic responses were noted at 0.25 mg/mL and 0.125 mg/mL, indicating that HTE triggers apoptosis even at lower therapeutic concentrations. The doxorubicin-treated group (DOX), serving as a positive control, showed the most pronounced apoptotic shift, although the effect of 1 mg/mL HTE approached a comparable level of efficacy. Quantitative analysis of the mean apoptotic populations (Figure 6B,C) confirmed these observations. Statistical analysis via one-way ANOVA followed by Tukey’s post hoc test revealed significant differences across all treatment conditions compared to the control. The percentage of live cells decreased progressively with increasing HTE concentrations, while the total apoptotic population (early + late apoptosis) showed a significant dose-dependent increase (*p* < 0.0001). These findings demonstrate that HTE effectively induces apoptosis in MIA PaCa-2 cells, primarily by increasing the population of cells in the late apoptotic stage, thereby significantly reducing overall cell viability.

#### 2.5.5. Genotoxic Effects of *H. triquetrifolium* in MIA PaCa-2 and HDF Cells

Evaluation of genotoxic effects on MIA PaCa-2 cells exposed to varying concentrations of the plant extract revealed a significant increase in DNA damage with rising concentrations (Table 5). This concentration-dependent cytotoxicity is likely attributable to the genotoxic action of the extract, which mirrors the primary mechanism of many anticancer drugs—increased DNA damage [13]. These findings align with the results reported by Mohammed and Ali and Mahajna et al. [14,15], collectively supporting the efficacy of the plant extract against pancreatic cancer. Similarly, in HDF cells, *H. triquetrifolium* extract induced a concentration-dependent increase in genotoxicity, most pronounced at the highest concentration of 1 mg/mL. The DNA damage observed at these elevated concentrations can be attributed to the cytotoxic effects of the extract.

## 3. Discussion

The present study provides comprehensive evidence of the antioxidant, anti-inflammatory, and anticancer potential of HTE extract, demonstrating its ability to modulate oxidative stress, inflammatory responses, and cancer cell proliferation. Phytochemical characterization revealed the presence of major bioactive constituents, including rutin, hyperoside, chlorogenic acid, neochlorogenic acid and hypericin, which are known to exhibit strong antioxidant, anti-inflammatory, and cytotoxic properties. These findings are consistent with previous studies demonstrating that phenolic compounds in *Hypericum* species play a critical role in regulating cellular signaling pathways associated with oxidative stress, inflammation, and tumor progression [9,16,17]. Hypericin has been widely reported to induce apoptosis, inhibit angiogenesis, and suppress tumor growth in various cancer models, while hyperoside and chlorogenic acid exhibit well-documented antiproliferative and cytotoxic effects, further supporting the pharmacological relevance of HT extract [18].

Oxidative stress plays a central role in carcinogenesis through excessive generation of ROS, which promotes DNA damage, genomic instability, and tumor progression. The notable antioxidant capacity observed in the DPPH assay (205.23 ± 9.49 mg TE/g) suggests that the whole-plant ethanol extract of *Hypericum triquetrifolium* effectively neutralizes free radicals. When compared with previous studies on the same species, antioxidant capacity appears to vary considerably depending on the extraction solvent and plant part. Dall’Acqua et al. reported DPPH values ranging from 121.83 to 407.35 mg TE/g across different *H. triquetrifolium* extracts, with the highest activity observed in aqueous root and aerial part extracts [19]. In the same study, CUPRAC and FRAP values reached up to 694.90 mg TE/g and 434.76 mg TE/g, respectively, exceeding those obtained in the present study (297.65 ± 3.16 mg TE/g for CUPRAC and 158.26 ± 4.03 mg TE/g for FRAP). These differences likely arise from variations in extraction methods, solvent polarity, plant part selection, and environmental and geographical factors influencing phytochemical composition.

When compared with other *Hypericum* species, the antioxidant activity observed in the present study is lower than that reported for *H. perforatum*, which can reach approximately 681 mg TE/g in the DPPH assay [20], but remains comparable to or higher than that of species such as *H. montanum* and *H. pubescens* [20]. Comparative analyses across the genus indicate that antioxidant capacity varies widely, largely depending on phenolic composition and metabolite profiles. The relatively strong activity of *H. triquetrifolium* may be attributed to its phenolic content (267 mg GAE/g extract), which falls within the range reported for *H. perforatum* (201.4 ± 2.8 to 371 mg GAE/g, depending on extraction conditions) [20,21,22]. Given the established role of phenolic compounds, particularly flavonoids and phenolic acids, in radical scavenging, this comparable phenolic profile likely contributes to the observed antioxidant capacity. Although lower than that reported for highly active species such as *H. perforatum*, the extract still demonstrates notable free radical scavenging activity. Overall, these findings support the potential of *H. triquetrifolium* as a natural antioxidant source and highlight the influence of extraction conditions and phytochemical composition on bioactivity. Consistent with these findings, HTE significantly reduced intracellular ROS levels and lipid peroxidation in LPS-stimulated RAW264.7 cells, as demonstrated by decreased fluorescence intensity and reduced MDA levels. Compared with the reference compounds, L-NAME and indomethacin, HTE showed a pronounced reduction in ROS production under the tested experimental conditions, suggesting a broader mechanism of action beyond selective inhibition of nitric oxide synthase or cyclooxygenase pathways. The reduction in lipid peroxidation further indicates the protective role of HTE against oxidative membrane damage, a key contributor to inflammation-mediated carcinogenesis.

Chronic inflammation is closely associated with cancer development, as persistent production of inflammatory mediators promotes tumor growth, angiogenesis, and immune evasion. In the present study, HTE reduced key pro-inflammatory mediators, including NO, PGE_2_, and IL-6, in LPS-stimulated macrophages. The suppression of these mediators suggests that HTE may disrupt the inflammatory microenvironment that supports tumorigenesis. The anti-inflammatory findings particularly at higher extract concentrations should be evaluated in relation to reduced cellular viability and non-specific cytotoxic effects may partially contribute to the observed decreases in inflammatory mediators. Therefore, greater emphasis should be placed on the biological responses observed at non-cytotoxic concentrations, which are more likely to reflect specific anti-inflammatory activity. The observed anti-inflammatory activity, together with its antioxidant properties, indicates that HTE exerts a dual protective effect by reducing both oxidative stress and inflammation, two major factors that promote cancer progression.

The ability of HTE to regulate oxidative stress was further confirmed by quantitative analysis of intracellular ROS levels. LPS stimulation significantly increased ROS production, consistent with previous reports demonstrating that inflammatory stimuli promote oxidative stress through macrophage activation [23]. While L-NAME and indomethacin showed limited effects on ROS reduction due to their specific mechanisms targeting nitric oxide synthesis and cyclooxygenase activity, HTE markedly decreased fluorescence intensity, particularly at higher concentrations. This enhanced ROS suppression may be attributed to the combined action of multiple phenolic compounds present in the extract, which may act synergistically to enhance antioxidant capacity.

The anticancer potential of HTE was further supported by its selective cytotoxic effects against MIA PaCa-2 pancreatic cancer cells. The extract induced a dose-dependent reduction in cancer cell viability while exhibiting lower toxicity toward normal human dermal fibroblasts, indicating selective targeting of malignant cells. Selective cytotoxicity is a desirable characteristic for potential anticancer agents, as it minimizes damage to healthy tissues [24]. Furthermore, the reduction in spheroid size observed in three-dimensional cell culture models supports the ability of HTE to inhibit tumor growth under physiologically relevant conditions, providing stronger evidence for its therapeutic potential compared with conventional two-dimensional models.

Mechanistically, HTE interfered with cancer cell proliferation by inducing cell cycle arrest and apoptosis. Cell cycle analysis revealed a concentration-dependent accumulation of MIA PaCa-2 cells in the G0/G1 phase, suggesting inhibition of progression into the S phase and suppression of DNA synthesis. The strongest G1 arrest was observed at moderate concentrations, whereas higher concentrations appeared to induce generalized non-specific cytotoxic effects, possibly due to excessive cellular stress. Apoptosis analysis demonstrated a shift from viable to late apoptotic populations with increasing extract concentration, indicating activation of programmed cell death pathways. Although HTE reduced intracellular ROS levels, its pro-apoptotic effects may reflect complex regulatory processes beyond ROS modulation in cancer cell survival. At higher concentrations, the observed effects may reflect non-specific cytotoxicity associated with excessive cellular stress, whereas lower concentrations are more likely to represent biologically relevant responses [16,25].

Previous studies have similarly reported antiproliferative and pro-apoptotic effects of *Hypericum triquetrifolium* in various cancer models. In addition to recent findings, earlier studies have demonstrated that constituents of *H. triquetrifolium* exhibit cytotoxic and antioxidant-related bioactivities in cancer systems. For example, Conforti et al. reported cytotoxic activity of antioxidant constituents isolated from *H. triquetrifolium*, suggesting a potential role of its phytochemical composition in anticancer effects [26]. Similarly, Couladis et al. demonstrated antioxidant and cytotoxic activities of *Hypericum* species in both brine shrimp and human cancer cell line models, further supporting the biological activity of the genus [27]. Al Anee et al. reported cytotoxic and growth-inhibitory effects of *H. triquetrifolium* methanolic extract against several cancer cell lines, including HepG2, PC3, MDA, and A549 cells [28]. Mahajna et al. demonstrated significant apoptosis induction and G0/G1 cell cycle arrest in HCT-116 colon cancer cells treated with *H. triquetrifolium* extract. Annexin V analysis revealed a strong dose-dependent increase in apoptotic cell populations, reaching up to 96% at higher concentrations. In addition, caspase-3 activation and suppression of cell cycle progression machinery further supported its pro-apoptotic and antiproliferative effects [15].

The anticancer potential of *Hypericum* species in pancreatic cancer has also been supported by both extract-based and isolated-compound studies. Saç et al. showed that *Hypericum perforatum* extract induced dose-dependent cytotoxicity in AR42J pancreatic cancer cells, accompanied by nuclear morphological changes consistent with apoptotic cell death [29]. Wang et al. isolated polyprenylated acylphloroglucinols from *Hypericum sampsonii* that exhibited cytotoxic activity against six pancreatic carcinoma cell lines, further supporting the pancreatic anticancer relevance of the genus [30]. Likewise, Li et al. reported that hyperoside inhibited tumor growth and induced apoptosis in pancreatic cancer models both in vitro and in vivo through modulation of Bcl-2 family proteins and NF-κB signaling [31].

Previous studies have suggested that *Hypericum* species may influence signaling pathways involved in cancer progression, including NF-κB and MAPK pathways [16,25]. However, as these pathways were not evaluated in the present study, their possible involvement in HTE-induced effects remains to be elucidated. Collectively, these findings support the anticancer potential of HTE, although further studies are required to clarify its molecular mechanisms in pancreatic cancer models.

When interpreted together with previous reports on *Hypericum triquetrifolium* and related *Hypericum* species, the present findings suggest that the observed biological activities are likely associated with the rich phenolic profile of the extract rather than a single constituent alone. Similar antioxidant, anti-inflammatory, and antiproliferative effects have been reported for several *Hypericum* species, particularly those rich in flavonoids and phenolic acids, supporting the view that these metabolites contribute to modulation of oxidative stress and inflammatory signaling pathways. In this context, the demonstrated reduction in intracellular ROS levels, inflammatory mediators, and pancreatic cancer cell proliferation is in line with the broader pharmacological profile previously described for the genus. However, differences in extraction solvent, plant part used, geographic origin, and phytochemical composition among studies may substantially influence potency and biological responses, which should be considered when comparing results across the literature. Therefore, the current data extend existing knowledge by providing evidence for the bioactivity of *Hypericum triquetrifolium* while also highlighting the need for standardized extracts and mechanistic studies to better define its therapeutic relevance.

The present findings contribute to the limited literature on *Hypericum triquetrifolium* by demonstrating its multifunctional biological activity within a single experimental framework. Overall, these results support the relevance of this species in pharmacology, oncology, and natural product research, particularly as a potential source of bioactive compounds targeting oxidative stress, inflammation, and cancer-related pathways [32,33,34].

## 4. Materials and Methods

### 4.1. Plant Material

The plant was collected from Çanakkale in Türkiye (Eceabat, Arıburnu, Anzak Koyu; Balıkçı Damları) on 27 July 2024. Plant identification was led by Prof. Dr. Ersin Karabacak (Department of Biology, Faculty of Science, Çanakkale Onsekiz Mart University, Çanakkale, Türkiye) and examined by Dr. Mehmet Ali Oçkun (Yeditepe University, Faculty of Pharmacy, İstanbul, Türkiye). The voucher specimen of *H. triquetrifolium* (YEF 21051) has been deposited at the Herbarium of the Department of Pharmacognosy, Faculty of Pharmacy, Yeditepe University, İstanbul, Türkiye. The above-ground parts (stem, leaves, and flowers) were air-dried and stored at room temperature.

### 4.2. Extraction

The *Hypericum* genus contains photosensitive compounds; therefore, all steps were performed under light protection. Ultrasonication, one of the most commonly used extraction techniques, was applied in this study to obtain the hydroalcoholic extract [35]. Prior to extraction, the aerial parts of the plant (equal quantities from each part: stem, leaves, and flowers) were air-dried and ground into a fine powder. A total of 3 g of powdered material was extracted with 30 mL of ethanol–water (80:20, *v*/*v*) and sonicated for 45 min. Following the centrifugation at 3500 rpm for 30 min., the supernatant was filtered, and the solvent was evaporated using a rotary evaporator. The extract was stored in amber glass bottles at 4 °C in the dark until analysis. The extraction yield was 40%.

### 4.3. Phytochemical Characterization

#### HPTLC Characterization of *H. triquetrifolium*

***Preparation of the Sample Test Solution***

The extract was re-dissolved in the solvent mixture containing ethanol–water (80:20, *v*/*v*) to obtain a stock solution (100 mg/mL), which was further diluted to 10 mg/mL for HPTLC analysis.

***Preparation of the Standard Solution***

The standard solutions for HPTLC analysis comprised the following compounds: rutin, chlorogenic acid, neochlorogenic acid, hyperoside, and hypericin. Chlorogenic acid and neochlorogenic acid were prepared at a concentration of 100 µg/mL, while rutin and hypericin were prepared at a concentration of 150 µg/mL. Hypericin was prepared at a concentration of 5 µg/mL. All standard solutions were prepared with methanol.

***HPTLC Method***

The sample test solution (10 mg/mL, 2 µL) and standard solutions (2 µL) were applied on glass-backed HPTLC silica gel 60 F_245_ plate (20 × 10 cm) using an automatic TLC sampler (ATS4, Camag, Muttenz, Switzerland). The HPTLC plate was developed to 7 cm in a saturated (20 min) twin-trough chamber using a developing solvent system of ethyl acetate–acetic acid–formic acid–water (100:11:11:26, *v*/*v*/*v*/*v*). After development, the plate was dried using cold air. Before derivatization, it was heated on a TLC plate heater (Camag) at 100 °C for 3 min. After that, the plate was first dipped into Naturstoff reagent A and then into polyethylene glycol 400 [36]. Finally, the plate was visualized at 366 nm using a Visualiser (Camag). Data acquisition and analysis were performed using VisionCATS v3.2. software.

### 4.4. Determination of Total Phenolic Content

A 25 µL aliquot of sample, standard (gallic acid), or distilled water (blank) was added to a 96-well microplate, followed by 10% diluted Folin reagent and 7.5% sodium carbonate. After incubation at 37 °C for 30 min, absorbance was measured at 760 nm. The results were expressed as mg of gallic equivalents (GAE) per g of hydroalcoholic extract (mg GAE/g extract) [37].

### 4.5. Determination of Antioxidant Properties

The results of antioxidant analyses performed in this study were expressed as mg of trolox equivalents (TE) per g of hydroalcoholic extract (mg TE/g hydroalcoholic extract).

#### 4.5.1. DPPH (2,2-diphenyl-1-picrylhydrazyl) Free Radical Scavenging Assay

In 96-well microplates, 20 μL of the sample test solution, trolox standard solutions at different concentrations, or methanol (blank) was added. Subsequently, 280 µL of a 0.1 mM methanolic DPPH solution was added, and the mixture was incubated at room temperature in the dark for 30 min before measuring at 530 nm [38].

#### 4.5.2. Cupric Ion-Reducing Antioxidant Capacity (CUPRAC) Assay

The CUPRAC method was applied as described by Apak et al. (2004) [39]. In each well of a 96-well microplate, 43 µL of the sample test solution or trolox standard solutions (3.125–200 μg/mL) or water as a blank was added. Then, 85 µL of 10 mM copper (II) sulfate pentahydrate, 7.5 mM neocuproine, ammonium acetate buffer (pH 7), and 51 µL of distilled water were added sequentially. After a 30 min incubation at room temperature, the absorbance was measured at 450 nm.

#### 4.5.3. FRAP (Ferric Reducing Antioxidant Power) Assay

The FRAP solution was prepared by mixing one volume of iron (III) chloride solution, one volume of tripyridyltriazine solution, and ten volumes of sodium acetate buffer (pH 3.6). After adding 20 µL of the sample, the test solution or trolox standard solutions (3.125–200 μg/mL) or blank, 280 µL of prepared FRAP reagent was added to each well of a 96-well microplate. After 6 min, the absorbance was measured at 595 nm [40].

### 4.6. Evaluation of Protective Effect Against Inflammation, Oxidative Stress and Cancer

#### 4.6.1. Cytotoxicity Assay on RAW264.7 Cell Line

The RAW264.7 murine macrophage cell line (ATCC, Manassas, VA, USA) was cultured in DMEM (Gibco, Paisley, UK) supplemented with 10% FBS, streptomycin (10,000 μg/mL), and 1% penicillin (10,000 units/mL) in a humidified atmosphere at 37 °C with 5% CO_2_. Cell viability was assessed using the MTT colorimetric assay. Plated RAW 264.7 cells were treated with various concentrations of the sample test solution of *H. triquetrifolium* extract (1.5–1000 μg/mL). After 24 h, the culture medium was removed. MTT solution (0.5 mg/mL) was added to the wells and incubated at 37 °C for another 2 h [41]. After incubation, the culture medium was removed, and 100 μL isopropanol was added to each well to dissolve the formazan crystals. Absorbance was measured at 570 nm using a microplate reader (Thermo Multiskan Spectrum, Helsinki, Finland). The percentage of cell viability was calculated using the following equation:

*Cell viability* (%) = (*OD*_570_ (*sample*)/*OD*_570_ (*medium control*)) × 100 (*OD*: *Optical Density*) (1)

#### 4.6.2. Effect of *H. triquetrifolium* on NO Production in RAW264.7 Cells

The anti-inflammatory activity of *H. triquetrifolium* extract was evaluated by measuring the levels of the stable nitric oxide (NO) metabolite, nitrite, in the cell culture medium using Griess reagent [42]. RAW264.7 cells were seeded in a 96-well plate and incubated at 37 °C with 5% CO_2_ for 24 h. Cells were pre-treated with *H. triquetrifolium* extract for 2 h prior to 1 μg/mL LPS (lipopolysaccharide derived from *Escherichia coli* 0111: B4, Sigma, Ronkonkoma, NY, USA) stimulation and subsequently co-incubated with LPS for an additional 22 h. The collected culture supernatants were mixed with Griess reagent (0.1% N-(1-naphthyl) ethylenediamine dihydrochloride in 5% phosphoric acid and 1% sulfanilamide) and incubated in the dark at room temperature for 10 min. Absorbance was measured at 540 nm using a microplate reader (Multiskan Ascent, Vantaa, Finland). Nitrite concentration was determined using a sodium nitrite standard curve. Indomethacin (100 μM) was used as a positive control [43].

#### 4.6.3. Anti-Inflammatory Activity via PGE_2_ Inhibition Assay

Concentrations showing significant anti-inflammatory activity were used in evaluating anti-inflammatory activity. The levels of prostaglandin E_2_ (PGE_2_) in the collected cell culture supernatants were detected using a quantitative enzyme-linked immunosorbent assay (ELISA) kit (Abcam PGE_2_ ELISA Kit, Cambridge, UK) according to the manufacturer’s instructions [44].

#### 4.6.4. IL-6 Inhibition Assay

The anti-inflammatory effect was further evaluated by measuring IL-6 levels using an enzyme-linked immunosorbent assay (ELISA). IL-6 is a key pro-inflammatory cytokine that is markedly elevated during inflammatory responses and represents an important target for therapeutic agent intervention [45]. IL-6 levels were quantified using a commercially available ELISA kit (eBioscience High-Performance Immune Assay, Vienna, Austria) according to the manufacturer’s instructions [46] using spectrophotometry (Thermo Multiskan Spectrum, Finland). All results were expressed as the mean ± standard deviation (SD).

#### 4.6.5. Assessment of Lipid Peroxidation in RAW264.7 Cells

Malondialdehyde (MDA) levels, an indicator of lipid peroxidation, were determined based on the reaction with thiobarbituric acid (TBA) to form a colored complex measured at 532 nm [47]. Tetramethoxypropane (TMP) was used as a standard, and the results were expressed as nmol/g protein [48]. RAW264.7 cells were seeded in T-25 flasks and treated with different concentrations of *H. triquetrifolium* hydroalcoholic extract for 2 h, prior to 1 μg/mL LPS (lipopolysaccharide derived from *Escherichia coli* 0111: B4, Sigma, USA) stimulation and subsequently co-incubated with LPS for an additional 22 h. After treatment, cells were washed with PBS, trypsinized, centrifuged, and homogenized in PBS. Cell lysates were centrifuged, and the supernatants were collected for MDA analysis. Cell homogenates or standard was mixed with TBA reagent, incubated in a hot water bath, cooled to room temperature, and absorbance was measured at 532 nm using a UV spectrophotometer (Thermo Multiskan Spectrum, Finland). Distilled water was used as a blank. The results were expressed as mean values in nmol/g protein [49].

#### 4.6.6. Effect of HTE on ROS Production in LPS-Induced RAW264.7 Cells

Intracellular ROS levels were determined using a DCFDA/H_2_DCFDA Cellular ROS Assay Kit (Abcam, Cambridge, UK; ab113851) according to the manufacturer’s instructions. Briefly, cells were washed twice with 1× buffer and incubated with DCFDA reagent at 37 °C for 45 min in the dark [50]. Following incubation, the staining solution was removed, and the cells were washed again with 1× buffer. Fluorescence images were acquired from four randomly selected fields per well at 10× magnification using a Cytell Imaging System (GE Healthcare, Chicago, IL, USA/Image Solutions, Torrance, CA, USA). All experiments were performed in triplicate to ensure reproducibility. Fluorescence intensity was quantified using ImageJ software (NIH, Bethesda, MD, USA) to evaluate the relative ROS production across experimental groups.

#### 4.6.7. Evaluation of Potential Anticancer Activity

HDF (healthy human dermal fibroblast) cells (ATCC, USA) and MIA PaCa-2 human pancreatic cancer cells (ATCC, USA) were cultured in DMEM (Gibco, UK) supplemented with 10% FBS and 1% penicillin/streptomycin at 37 °C in a humidified atmosphere containing 5% CO_2_. Cytotoxicity of *H. triquetrifolium* extract was evaluated by using the MTT assay at different concentrations (0.0625–1 mg/mL) according to the method described above [51]. After treatment, cell viability was determined by measuring absorbance at 570 nm using a microplate reader. Cell viability was calculated relative to the control group, and concentrations resulting in cell viability below 70% were considered cytotoxic. Non-cytotoxic concentrations were used in subsequent experiments.

#### 4.6.8. Three-Dimensional (3D) Spheroid Formation/Growth Assay

For 3D spheroid formation, the MIA PaCa-2 cells and HDF cells were used according to the magnetic 3D Bioprinting method [52]. Human fibroblasts were mixed in equal numbers with pancreatic cancer cells to better mimic the tumor environment. After cells reached 70–80% confluency, cells were treated with biocompatible NanoShuttleTM-PL (Bioprinting Kit, Greiner Bio-One, Kremsmünster, Austria) and incubated overnight. After the incubation period, cells (2000 pancreatic cancer cells and 2000 human fibroblasts per well) were resuspended and seeded into ultra-low-attachment 96-well culture plates with a 100 μL volume [38]. Until spheroids were formed, the culture plate was placed on a magnetic drive and incubated in a humidified atmosphere containing 5% CO_2_ at 37 °C for 48 h [52]. Two days after the initial incubation period, the spheroids were photographed using light microscopy and subsequently treated with *H. triquetrifolium* test solution with concentrations of 0.0625–1 mg/mL. Over the following ten days, the treatment medium was refreshed every three days, during which photographs of the spheroids were taken at the same intervals. The effects of the extract on spheroid growth were evaluated by measuring changes in spheroid size using ImageJ software, with doxorubicin (DOX) with the concentration range of 1–10 µM serving as the reference compound.

#### 4.6.9. Cell Cycle Analysis of HTE-Treated MIA PaCa-2 Cells

Cell cycle distribution was analyzed using the Muse Cell Cycle Kit (Cytek Biosciences, Fremont, CA, USA; MCH100106) according to the manufacturer’s protocol. Briefly, MIA PaCa-2 cells were harvested, and 200 µL of the cell suspension was centrifuged at 300× *g* for 5 min. After discarding the supernatant, the cell pellet was washed once with PBS and fixed by the dropwise addition of 200 µL of ice-cold 70% ethanol, followed by incubation at −20 °C for at least 3 h. Prior to staining, cells were centrifuged again at 300× *g* for 5 min; ethanol was removed, and the pellet was washed once more with PBS. Finally, the fixed cells were resuspended in 200 µL of Muse Cell Cycle Reagent, incubated in the dark at room temperature for 30 min, and analyzed using the Muse Cell Analyzer (Cytek Biosciences). All experiments were conducted in triplicate.

#### 4.6.10. Apoptosis Analysis of HTE-treated MIA PaCa-2 cells

The pro-apoptotic effect of HTE on MIA PaCa-2 cells was evaluated using the Muse Annexin V & Cell Dead Kit (Cytek Biosciences, Fremont, CA, USA; MCH100105) according to the manufacturer’s protocol. Following the treatment period, both floating and adherent cells were collected and centrifuged at 300× *g* for 5 min. The supernatant was discarded, and the cell pellet was washed once with PBS. The cells were then resuspended, and the concentration was adjusted to 1 × 10^5^ cells/mL in 24-well plates. A 100 µL of cell suspension was mixed with 100 µL of the Muse Annexin V & Cell Dead reagent. The samples were gently pipetted and incubated for 20 min at room temperature in the dark. Data acquisition was performed using the Muse Cell Analyzer to assess the percentages of live, early apoptotic, late apoptotic, and dead cells. All assays were performed in triplicate.

#### 4.6.11. Single-Cell Gel Electrophoresis (COMET) Assay

MIA PaCa-2 and HDF cells were cultured in 25 cm^2^ flasks and trypsinized; they were then seeded into 24-well plates. The plates were incubated for 24 h at 37 °C in a 5% CO_2_ atmosphere with 95% humidity. Subsequently, the cells were exposed to cytotoxic concentrations of *H. triquetrifolium* extracts in serum-free DMEM (Gibco, UK). For the comet assay, the treated cells were mixed with normal melting agarose (NMA) and spread onto low-melting-point agarose (LMA)-coated slides, which were then kept at 4 °C for agar solidification. Next, the slides were incubated in lysis solution at 4 °C for at least 1 h. Following lysis, the slides were placed in an electrophoresis tank and equilibrated in electrophoresis buffer for 20 min. Electrophoresis was then performed at 4 °C using 24 V, with the current adjusted to 300 mA for 20 min. The slides were neutralized in PBS for 15 min, washed with distilled water, and sequentially dehydrated in 50%, 75%, and 98% ethanol. Finally, DNA damage was assessed by staining the slides with EtBr and analyzing 100 cells per slide using Comet Analysis Software version 4.0. The eesults were expressed as tail intensity [53].

### 4.7. Statistical Analysis

Cell viability (MTT), 3D spheroid growth, and comet assay data were analyzed using one-way ANOVA followed by Tukey’s post hoc test. Intracellular ROS production, cell cycle distribution, genotoxicity, and apoptosis levels were evaluated using one-way ANOVA followed by Tukey’s multiple comparison test, after confirming the parametric distribution of the data via the Shapiro–Wilk test. All analyses were performed using GraphPad Prism 10 (GraphPad Software, San Diego, CA, USA). Data are presented as the mean ± SD from three independent experiments.

## 5. Conclusions

This study provides preliminary evidence for the anticancer potential of *Hypericum triquetrifolium* in pancreatic cancer models, integrating antioxidant, anti-inflammatory, and antiproliferative effects. To our knowledge, this is the first study to comprehensively evaluate the extract in both 2D and 3D pancreatic cancer systems while simultaneously assessing its impact on oxidative stress, inflammatory mediators, cell cycle progression, and apoptosis.

The findings indicate that *H. triquetrifolium* modulates oxidative stress and inflammatory responses and inhibits cancer cell proliferation, with evidence of cell cycle arrest and apoptosis induction. The observed selective cytotoxicity toward cancer cells, relative to non-cancerous cells, further supports its biological relevance. However, these results should be interpreted with caution. Some effects observed at higher concentrations may be influenced by non-specific cytotoxicity associated with cellular stress. In addition, the absence of detailed phytochemical characterization (e.g., LC–MS analysis) limits the identification of the active constituents responsible for the observed effects. Overall, *H. triquetrifolium* represents a candidate of interest for further investigation, and additional in vitro and in vivo studies are required to validate these findings and elucidate the underlying molecular mechanisms.

## Figures and Tables

**Table 1:** Antioxidant activity of 0.2 mg/mL hydroalcoholic extract of *H. triquetrifolium*.

Sample	DPPH (mg TE/g) *	CUPRAC (mg TE/g) *	FRAP (mg TE/g) *
*H. triquetrifolium* (Aerial parts)-EtOH extract	205.23 ± 9.49	297.65 ± 3.16	158.26 ± 4.03

EtOH: Ethanolic; DPPH: 2,2-diphenyl-1-picrylhydrazyl; CUPRAC: cupric ion reducing antioxidant capacity; FRAP: Ferric Reducing Antioxidant Power; TE: Trolox equivalent. * The antioxidant activities were expressed as mg of trolox equivalents (TE) per g of hydroalcoholic extract (mg TE/g extract).

**Table 2:** Effects of *H. triquetrifolium* on viability and nitrite production in RAW264.7 cells.

Groups	Concentration	Cell Viability (%)	Nitrite Level (µM)	Nitrite Inhibition (%)
Control		115.93 ± 2.90	1.89 ± 1.57	-
LPS		100.00 ± 0.67	55.78 ± 1.31	-
L-NAME	100 µM	93.36 ± 1.27	29.30 ± 2.10 *	47.47
IND	100 µM	97.49 ± 1.29	27.07 ± 2.31 *	51.47
HTE (mg/mL)	0.0625	101.04 ± 1.78	23.49 ± 2.21 *	57.88
	0.125	91.30 ± 2.02	13.49 ± 2.53 *	75.81
	0.25	82.36 ± 1.57	9.91 ± 0.28	82.23
	0.5	74.19 ± 2.87	6.89 ± 0.93 *	87.65
	**1**	**33.63 ± 4.69**	-	-

Control: control group with DMEM; LPS: control group stimulated with LPS only; LPS: lipopolysaccharide from *E. coli*; IND: indomethacin (100 µM); L-NAME: N(gamma)-nitro-L-arginine methyl ester. Results shown are as mean values ± SD of three independent experiments. Statistically significant differences for each compound are indicated against LPS (* *p* < 0.05). Since values in bold indicate cell viability below 70%, nitrite levels and inhibition were not calculated.

**Table 3:** Effect of *H. triquetrifolium* extract on MDA levels in LPS-induced RAW264.7 cells.

Groups	Concentration (mg/mL)	MDA (nmol/g Protein)
Control	-	0.97 ± 0.28
LPS	-	2.59 ± 0.24
HTE	0.0626 0.125	1.37 ± 0.37 1.22 ± 0.34

Control: control group with DMEM; LPS: control group stimulated with LPS only; LPS: lipopolysaccharide from *E. coli*; HTE: *Hypericum triquetrifolium* extract; MDA: malondialdehyde.

**Table 4:** Effect of HTE on cell viability in HDF and MIA PaCa-2 cell lines.

			Cell Viability (%) *			
Groups (mg/mL)	0.0625	0.125	0.25	0.5	1	IC_50_
**HDF**	96.45 ± 6.44	86.79 ± 4.46	81.97 ± 4.01	71.49 ± 3.94	**13.55 ± 1.67**	0.68 ± 0.02
**MIA PaCa-2**	87.45 ± 5.28	**51.16 ± 10.01**	**49.16 ± 8.92**	**35.64 ± 10.07**	17.45 ± 2.46	0.18 ± 0.14

* Cell viability in the control group was 100.00% ± 2.19% in HDF cells and 100.00% ± 5.50% in MIA PaCa-2 cells. The percentage of cell viability in the treatment groups was calculated relative to the control group with DMEM. Values in bold indicate a significant reduction compared to the control group. HTE: *Hypericum triquetrifolium* extract; HDF: human dermal fibroblasts; MIA PaCa-2: human pancreatic carcinoma cell line; IC_50_: half-maximal inhibitory concentration.

**Table 5:** Effect of HTE extract on DNA damage in MIA PaCa-2 and HDF cell lines.

Groups	Concentration	Tail Intensity	
		MIA PaCa-2	HDF
NC	-	6.98 ± 0.39	9.30 ± 1.31
PC	25 µg/mL	48.90± 3.03 *	39.64 ± 4.48 *
HTE (mg/mL)	0.0625	7.04± 1.40	9.93 ± 2.34
	0.125	8.40± 0.40	11.40 ± 2.41
	0.25	11.33± 2.20	13.40 ± 1.98
	0.5	26.49± 3.39 *	14.49 ± 2.44
	1	41.98 ± 4.29 *	21.04 ± 3.41 *

* Statistically significant difference compared to negative control (*p* < 0.05). HTE: *H. triquetrifolium* extract. PC: positive control (25 mg/mL H_2_O_2_); NC: negative control; HTE: *Hypericum triquetrifolium* extract.

## Data Availability

The original contributions presented in this study are included in the article. Further inquiries can be directed to the corresponding author.
